# Effects of Decreased Immunization Coverage for Hepatitis B Virus Caused by COVID-19 in World Health Organization Western Pacific and African Regions, 2020

**DOI:** 10.3201/eid2813.212300

**Published:** 2022-12

**Authors:** Hyacinte J. Kabore, Xi Li, Robert D. Allison, Tigran Avagyan, Richard Mihigo, Yoshihiro Takashima, Rania A. Tohme

**Affiliations:** Centers for Disease Control and Prevention, Atlanta, Georgia, USA (H.J. Kabore, X. Li, R.D. Allison, R.A. Tohme);; World Health Organization, Brazzaville, Republic of the Congo (H.J. Kabore, R. Mihigo);; World Health Organization, Manila, Philippines (T. Avagyan, Y. Takashima)

**Keywords:** hepatitis B virus, COVID-19, respiratory infections, severe acute respiratory syndrome coronavirus 2, SARS-CoV-2, SARS, coronavirus disease, zoonoses, viruses, coronavirus, chronic hepatitis B, hepatitis B vaccines, health impact assessment, United States, Republic of the Congo, Philippines

## Abstract

The World Health Organization–designated Western Pacific Region (WPR) and African Region (AFR) have the highest number of chronic hepatitis B virus (HBV) infections worldwide. The COVID-19 pandemic has disrupted childhood immunization, threatening progress toward elimination of hepatitis B by 2030. We used a published mathematical model to estimate the number of expected and excess HBV infections and related deaths after 10% and 20% decreases in hepatitis B birth dose or third-dose hepatitis B vaccination coverage of children born in 2020 compared with prepandemic 2019 levels. Decreased vaccination coverage resulted in additional chronic HBV infections that were 36,342–395,594 in the WPR and 9,793–502,047 in the AFR; excess HBV-related deaths were 7,150–80,302 in the WPR and 1,177–67,727 in the AFR. These findings support the urgent need to sustain immunization services, implement catch-up vaccinations, and mitigate disruptions in hepatitis B vaccinations in future birth cohorts.

Chronic hepatitis B virus (HBV) infection is the leading cause of liver cancer and a major public health problem in the World Health Organization (WHO)–designated Western Pacific Region (WPR) and African Region (AFR). In 2019, of the estimated 296 million persons living with chronic HBV infection worldwide, ≈116 million (39%) resided in the WPR and ≈82 million (28%) resided in the AFR (*1*). Of the estimated 1.5 million new HBV infections that occurred globally, 140,000 (9%) new infections occurred in the WPR and 990,000 (66%) new infections occurred in the AFR (*1*).

In 2016, all WHO member states endorsed the global target for elimination of viral hepatitis as a public health threat by 2030; specific aims included reducing the prevalence of hepatitis B surface antigen (HBsAg), a marker of chronic HBV infection, to <0.1% among children 5 years of age and achieving >90% coverage with a hepatitis B vaccine birth dose (HepB-BD) and 3 additional infant doses (HepB3) (*2*). Interim targets to achieve viral hepatitis elimination by 2020 included HBsAg prevalence of <1% among children and coverage levels of >50% for HepB-BD and >90% for HepB3 among infants (*2*).

In both the WPR and AFR, all countries provide 3 doses of HBV vaccine; however, only 35 (95%) of 37 countries in the WPR and 13 (28%) of 47 countries in the AFR provide universal HepB-BD to all newborns. In addition, 2 countries in the WPR (Japan, New Zealand) and 1 country in the AFR (Mauritius) provide HepB-BD selectively to babies born to mothers who are HBsAg-positive. The WPR has made substantial progress in controlling HBV infection through successful HBV immunization programs for children. As of 2020, a total of 21 (57%) of 37 countries and areas in the WPR achieved HBsAg prevalence in <1% of children according to serosurvey evidence (*3*). The WPR target of >95% vaccination coverage was achieved by 19 (51%) WPR countries or areas for HepB-BD and 20 (54%) countries for HepB3, and the global target of >90% coverage was achieved by 23 (62%) countries for HepB-BD and 34 (92%) countries for HepB3 (*4*–*6*). However, progress toward hepatitis B control has been much slower in the AFR, where only 14 (30%) of the 47 countries have HepB-BD in their immunization schedule. Of those 14, only 5 (11%) countries reached the regional and global 2020 target of >50% HepB-BD coverage (*5*), and 19 (40%) of 47 countries achieved >90% HepB3 coverage (*6*). Nationally representative serosurvey data are lacking among children in most countries in Africa. However, modeled estimates reported that 25 (53%) countries achieved the regional target of <2% HBsAg prevalence and 13 (28%) countries achieved the global target of <1% HBsAg prevalence among children by 2020 (*7*).

The COVID-19 pandemic has strained public health capacity and disrupted the delivery and uptake of childhood vaccines, thereby threatening the control and elimination of major vaccine-preventable diseases. Of 61 countries worldwide that responded to a June 2020 immunization pulse poll, 45 (74%) countries reported a drop in vaccination demand, and the AFR reported the highest proportion of countries (89%) with decreased demand; in addition, 52 (85%) of responding countries reported a drop in coverage in May 2020 compared with January–February 2020 (*8*). Furthermore, vaccine shipments and supplies were affected early during the COVID-19 pandemic because of disruption in air transportation and closure of airports (*9*). As a result of COVID-19–related disruption of immunization services, ≈80 million children <1 year of age worldwide were at risk for vaccine-preventable diseases (*10*). In the WPR, preliminary data reported for the first quarter of 2020 showed a 10%–50% decrease in the number of children who completed 3 doses of HBV vaccine, and the average reduction in coverage was ≈20% (*11*). In the AFR, preliminary coverage data showed a drop in HepB3 coverage in 37 (79%) countries, and 10 countries reported a decrease in coverage >10% during the first half of 2020 compared with the same period in 2019 (*12*).

The COVID-19 pandemic and its negative effect on immunization and other essential health services poses a threat to progress toward decreasing the burden of hepatitis B among children and achieving HBV elimination by 2030. We estimated the additional numbers of chronic HBV infections and HBV-related deaths in the WPR and AFR, the regions most affected by HBV, that resulted from decreased HBV vaccination coverage during 2020 because of the COVID-19 pandemic.

## Materials and Methods

### Data Sources

We used a published mathematical model (*13*) to estimate the effects of decreased hepatitis B vaccination on HBV-related illness and death among children born in 2020. The model is a static model that estimates the number of HBV infections and deaths from mother-to-child transmission during the perinatal period (<1 year of age) and horizontal transmission during early (1–5 years of age) and late (>5 years of age) childhood. The model included the number of surviving infants, vaccination coverage with HepB-BD and complete HepB3 series, prevalence of HBsAg and HBV e antigen among women of reproductive age, and hepatitis B core antigen antibody prevalence among children 5 years of age and adults 30 years of age. The frequency of HBV seromarkers was compiled from published systematic reviews, population-based HIV impact assessments that included hepatitis B seroprevalence in AFR countries, and HBV profiles for WPR countries, which included nationwide and large-scale subnational serosurveys for the region. When data were not available from these sources, published estimates were used (Appendix Table 1). The efficacy of complete hepatitis B vaccination (HepB-BD and HepB3) was estimated to be 95% (*14*). For countries that did not provide HepB-BD but provided HepB3 vaccines as part of combination vaccines during childhood, we considered infants who received HepB3 vaccines to be unprotected against vertical transmission. However, because of the receipt of the 3 primary vaccines, we considered these infants to be protected against horizontal transmission with 95% vaccine efficacy (*14*). 

We obtained population and death rate data from World Population Prospects, 2019 revision, published by the United Nations Population Division (*15*). We compiled HBV vaccination coverage data from the 2019 WHO/UNICEF estimates of national immunization coverage (WUENIC) (*16*). WUENIC estimates were based on official or administrative survey coverage data and included contextual information, such as status of vaccine stock and changes in vaccination schedule. When WUENIC estimates were unavailable, we used official or administrative vaccination coverage reported in the WHO-UNICEF Joint Reporting Form. Administrative vaccination coverage data are derived from the country’s immunization registry system and may be inaccurate because of underestimates or overestimates; thus, national authorities can provide their official vaccination coverage estimates, which are based on administrative data, surveys, and reports. If WUENIC estimates were unavailable for HepB3, we used official estimates, and, if those were unavailable, we used administrative coverage estimates. If WUENIC estimates were unavailable for HepB-BD, we used data sources in this order of preference: official coverage estimates of birth doses administered within 24 h after birth; administrative coverage of birth doses given within 24 h after birth; official coverage of total birth doses, which included birth doses provided <24 h or >24 h after birth; and administrative coverage of total birth doses. When the reported coverage was >100%, the coverage was capped at 100%. For countries that had not introduced HepB-BD or countries that provided selective HepB-BD vaccines, coverage was not reported to WHO and we could not compute the effects of changes in HepB-BD coverage in those countries.

### Data Analysis

We estimated the total number of expected chronic HBV infections and deaths during the lifetime of children born in 2020 by first assuming HepB-BD and HepB3 coverage levels were identical to those in 2019, before the COVID-19 pandemic. We included deaths from HBV-related liver cirrhosis, hepatocellular carcinoma, and fulminant hepatitis in the analysis. A 20% reduction in vaccination coverage was reported in WPR countries in the first quarter of 2020 compared with 2019 (*11*), and a >10% decline in vaccination coverage was observed in the AFR during the first 6 months of 2020 compared with 2019 (*12*). Therefore, we estimated the number of chronic HBV infections and deaths in children born in 2020 when HepB-BD or HepB3 was decreased by 10% and 20% in 2020 compared with 2019.

For the AFR, we analyzed the number of chronic HBV infections and related deaths according to HepB-BD introduction status in countries from 3 operational geographic areas: Central Africa (10 countries), East/southern Africa (20 countries), and West Africa (17 countries). We calculated numbers of excess chronic HBV infections and related deaths on the basis of the estimated 10% or 20% decline in HepB-BD and HepB3 coverage in 2020 compared with 2019.

## Results

**Figure 1:**
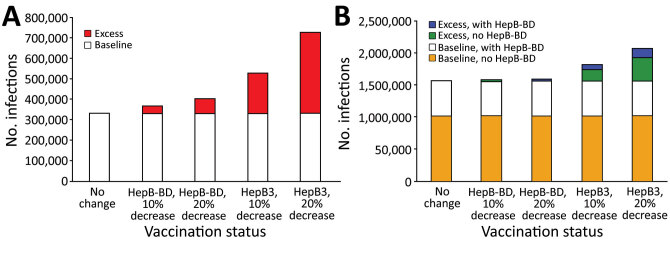
Numbers of additional chronic hepatitis B cases after decreased coverage for hepatitis B vaccine caused by COVID-19 in World Health Organization (WHO) Western Pacific Region (WPR) and African Region (AFR), 2020. We used a mathematical model to estimate the effect of decreased hepatitis B vaccination coverage on hepatitis B virus (HBV) infections among children born in 2020 compared with 2019. A) Total number of chronic HBV infections determined from 2019 data (baseline) and estimates of excess chronic HBV infections from the model after 10% or 20% decrease in HepB-BD or HepB3 vaccination coverage in the World Health Organization WHO Western Pacific Region. All countries and areas in the WPR have introduced HepB-BD, including 2 countries that provide HepB-BD only to infants born to hepatitis B surface antigen–positive mothers. B) Total number of chronic HBV infections (baseline) and estimates of excess chronic HBV infections after 10% or 20% decrease in HepB-BD or HepB3 vaccination coverage in the WHO AFR. Comparisons were made between countries with and without HepB-BD. Fourteen countries in the AFR have introduced HepB-BD, including 1 country that provides HepB BD-only to infants born to hepatitis B surface antigen–positive mothers. HepB-BD coverage data were only available for countries that provided universal birth doses. HepB-BD, birth dose; HepB3, third-dose hepatitis B.

We used the model to estimate the number of chronic HBV infections and HBV-related deaths during the lifetime of children born in 2020 who received different HBV vaccination coverage (Table). If 2019 hepatitis B vaccination coverages were maintained in 2020, the model estimated 332,179 chronic HBV infections in the WPR and 1,564,688 chronic HBV infections in the AFR during the lifetime of children born in 2020. If either HepB-BD or HepB3 coverage dropped by 10% or 20% in 2020 compared with 2019, the total estimated numbers of chronic HBV infection in these children would be 368,521–727,773 in the WPR and 1,574,481–2,066,735 in the AFR (Table). The number of additional chronic HBV infections ranged from 36,342 (11%) to 395,594 (119%) in the WPR (Figure 1, panel A) and 9,793 (1%) to 502,047(32%) in the AFR (Figure 1, panel B). In AFR countries that introduced HepB-BD, a 10% decrease in HepB3 coverage from baseline resulted in 70,225 (13%) additional chronic HBV infections, whereas a 10% decrease in HepB-BD coverage resulted in an estimated 9,792 (2%) excess chronic HBV infections (Figure 1, panel B). In AFR countries that did not provide HepB-BD, a 10% decrease in HepB3 coverage caused 180,798 (18%) excess chronic HBV infections.

**Table:** Estimated numbers of chronic hepatitis B virus infections and related deaths after decreased hepatitis B vaccine birth dose and third dose vaccination coverage caused by COVID-19 in World Health Organization Western Pacific and African Regions, 2020*

Variables	Baseline, 2019	Hepatitis B birth dose			Hepatitis B third-dose†	
		Decrease, 10%	Decrease, 20%		Decrease, 10%	Decrease, 20%
Western Pacific Region						
Hepatitis B birth dose coverage	84%	76%	67%		NA	NA
Hepatitis B third-dose coverage	94%	NA	NA		85%	75%
Chronic infections	332,179	368,521	404,863		529,976	727,773
HBV-related deaths						
Hepatocellular carcinoma	29,779	33,392	37,004		49,394	69,009
Liver cirrhosis	31,002	34,538	38,075		50,589	70,175
Fulminant hepatitis	1,442	1,442	1,442		2,390	3,339
Total	62,222	69,372	76,521		102,373	142,524
African Region						
Hepatitis B birth dose coverage	17%	15%	14%		NA	NA
Hepatitis B third-dose coverage	73%	NA	NA		66%	58%
Chronic infections	1,564,688	1,574,481	1,584,273		1,815,712	2,066,735
HBV-related deaths						
Hepatocellular carcinoma	81,267	81,805	82,342		94,405	107,543
Liver cirrhosis	112,011	112,651	113,290		130,895	149,779
Fulminant hepatitis	7,891	7,891	7,891		9,733	11,575
Total	201,170	202,347	203,524		235,033	268,897

*We assumed that in the absence of the COVID-19 pandemic, 2019 hepatitis B vaccination coverage levels would have been maintained in 2020; hepatitis B vaccination coverages for 2020 were estimated from 2019 baseline coverage levels, and baseline coverage levels were from World Health Organization/UNICEF estimates of national immunization coverage or derived from official or administrative estimates from specific countries. HBV, hepatitis B virus; NA, not applicable. †Hepatitis B third-dose coverage was a combination of a hepatitis B birth dose and 2 additional doses of hepatitis B vaccine in 14 countries in the Western Pacific Region. Other countries provided up to 3 additional hepatitis B doses in a pentavalent vaccine.

**Figure 2:**
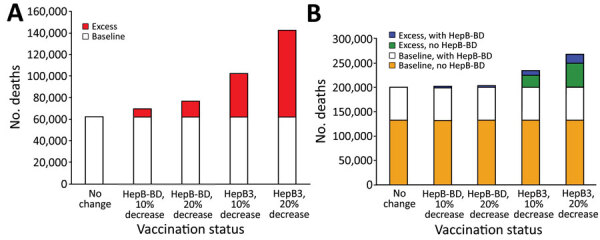
Numbers of additional hepatitis B virus (HBV)–related deaths after decreased coverage for hepatitis B vaccine caused by COVID-19 in World Health Organization (WHO) Western Pacific Region (WPR) and African Region (AFR), 2020. We used a mathematical model to estimate the effect of decreased hepatitis B vaccination coverage on HBV-related deaths among children born in 2020 compared with 2019. A) Total number of HBV-related deaths determined from 2019 data (baseline) and estimates of excess deaths after 10% or 20% decrease in birth dose (HepB-BD) or third-dose hepatitis B (HepB3) vaccination coverage in the WHO WPR. All countries and areas in the WPR have introduced HepB-BD, including 2 countries that provide HepB-BD only to infants born to hepatitis B surface antigen–positive mothers. B) Total number of HBV-related deaths (baseline) and estimates of excess deaths after 10% or 20% decrease in HepB-BD or HepB3 vaccination coverage in the WHO AFR. Comparisons were made between countries with and without HepB-BD. Fourteen countries in the AFR have introduced HepB-BD, including 1 country that provides HepB BD-only to infants born to hepatitis B surface antigen–positive mothers. HepB-BD coverage data were only available for countries providing universal birth doses. HepB-BD, birth dose; HepB3, third-dose hepatitis B.

If 2019 levels of HBV vaccination coverage were maintained in 2020, the model estimated that HBV infections produced 62,222 HBV-related deaths in the WPR and ≈201,170 HBV-related deaths in the AFR (Table). If either HepB-BD or HepB3 decreased by 10% or 20% in 2020, the total number of HBV-related deaths would be 69,372–142,524 in the WPR and 202,347–268,897 in the AFR (Table) during the lifetime of children born in 2020. The increases in HBV-related deaths were from 7,150 (11%) to 80,302 (129%) in the WPR (Figure 2, panel A) and 1,177 (1%) to 67,727 (34%) in the AFR (Figure 2, panel B) compared with baseline values. In AFR countries that provided HepB-BD, a 10% decrease in HepB3 coverage resulted in 9,499 (14%) additional deaths, whereas a 10% decrease in HepB-BD coverage caused 1,177 (2%) excess deaths (Figure 2, panel B). In AFR countries that did not provide HepB-BD, a 10% decrease in HepB3 coverage produced 24,365 (18%) excess HBV-related deaths.

We estimated total lifetime numbers of chronic HBV infections and deaths among children born in 2020 by country for each region (Appendix Tables 2, 3). If 2019 hepatitis B vaccination coverage levels were maintained in the WPR in 2020, the highest estimated numbers of chronic HBV infections would have occurred in China (123,186), the Philippines (122,717), Vietnam (40,359), and Papua New Guinea (20, 888) and accounted for 307,150 (92%) of all 332,179 expected chronic infections in this region (Appendix Table 2). A 10% decrease in HepB3 coverage produced a 155% increase in chronic HBV infections in Brunei Darussalam, 131% increase in Fiji, and 131% increase in China. A 20% decrease in HepB3 coverage produced a 310% increase in chronic HBV infections in Brunei Darussalam, 265% increase in Fiji, and 256% increase in China. A 10% decrease in HepB-BD coverage resulted in an 80% increase in chronic HBV infections in Tonga, 32% increase in Mongolia, and 27% increase in Fiji; a 20% decrease in HepB-BD coverage caused a159% increase in infections in Tonga, 64% increase in Mongolia, and 54% increase in Fiji (Appendix Table 2).

If 2019 HBV vaccination coverage levels were maintained in the AFR in 2020, countries in West Africa accounted for the highest number of chronic HBV infections (675,017), followed by East/southern Africa (463,185) and Central Africa (426,486) (Appendix Table 3). A 10% decrease in HepB3 coverage produced a 25% increase in chronic infections in countries in East/southern Africa, 14% increase in West Africa, and 9% increase in Central Africa. We estimated that the highest number of expected chronic HBV infections were in Nigeria (384,442), the Democratic Republic of the Congo (227,219), and Ethiopia (150,025) (Appendix Table 3). A 10% decrease in HepB3 coverage produced a 100% increase in chronic HBV infections in Rwanda, 93% increase in Cabo Verde, 75% increase in Sao Tome and Principe, and 63% increase in Algeria. A 20% decrease in HepB3 coverage caused a 199% increase in chronic HBV infections in Rwanda, 186% increase in Cabo Verde, 149% increase in Sao Tome and Principe, and 125% increase in Algeria. Among AFR countries that provided HepB-BD vaccination, a 10% decrease in HepB-BD coverage resulted in a 21% increase in excess chronic HBV infections in Cabo Verde, an 18% increase in Sao Tome and Principe, 11% increase in Senegal, and 10% increase in Botswana. A 20% decrease in HepB-BD coverage produced a 43% increase in excess chronic HBV infections in Cabo Verde, 36% increase in Sao Tome and Principe, 21% increase in Senegal, and 36% increase in Botswana (Appendix Table 3). Estimated increases in HBV-related deaths after decreased HepB3 or HepB-BD coverage showed patterns similar to those of chronic HBV infections in countries from the WPR and AFR (Appendix Tables 2, 3).

## Discussion

The COVID-19 pandemic has led to substantial disruptions in routine immunization services and subsequent reductions in vaccination coverage in the WPR and AFR. Using a mathematical model (*13*), we estimated that a 20% decrease in HepB3 vaccination coverage because of COVID-19 would produce >500,000 excess chronic HBV infections and >67,000 additional HBV-related deaths in the AFR and >395,000 excess chronic infections and >80,000 additional HBV-related deaths in the WPR during the lifetimes of children born in 2020. A 10% decrease in HepB3 vaccinations also would produce substantial increases in chronic HBV infections and HBV-related deaths in both the AFR and WPR. A 10% decrease in HepB-BD vaccination coverage in the WPR would produce an estimated 11% increase in chronic HBV infections or HBV-related deaths compared with a 1% increase in chronic infections or HBV-related deaths in the AFR. This difference is likely because the AFR had a low baseline HepB-BD coverage of only 15% compared with 84% HepB-BD coverage in the WPR in 2019 (*17*). However, the increase in chronic HBV infections after a 10% decrease in HepB3 coverage was 1.4 times lower in AFR countries that introduced HepB-BD vaccinations compared with countries without HepB-BD, which indicates the value of HepB-BD vaccination in lowering HBV infection rates. As recently reported, wider introduction of HepB-BD in the AFR will enhance progress toward HBV elimination and prevent further infections and deaths (*18*).

The effects of COVID-19–related disruptions to immunization services may not be limited to children born in 2020. After the initial COVID-19 wave during January–February 2020, both the WPR and AFR experienced subsequent waves during July–December 2020 (*19*). Because of emerging new SARS-CoV-2 variants, spikes in COVID-19 cases are expected to continue (*9*). A pulse survey on continuity of essential health services was conducted in 2021 and, among countries responding to the survey, 24% of countries in the WPR and 48% in the AFR reported ongoing disruptions in immunization services (*20*). 

Childhood immunization is recognized as a core health service that should continue during the COVID-19 pandemic in conjunction with COVID-19 prevention and control measures for caregivers and health workers (*21*). Administering the HepB-BD vaccine within 24 hours of birth prevents 70%–95% of perinatal transmission from HBV-infected mothers (*22*). In the WPR, >90% of births are hospital-based, and neonates are more likely to receive HepB-BD vaccination when born in hospitals (*23*). However, the pulse survey indicated that 20% of WPR countries had disruptions in facility-based births, and 43% of countries reported disruptions in antenatal but not postnatal care (*20*). Therefore, hospitals in the WPR should include HepB-BD vaccinations in their COVID-19 prevention and control planning and protocols to ensure newborns continue to receive HepB-BD within 24 hours after birth during the COVID-19 pandemic. Disruptions in facility-based births were reported in 31% of AFR countries; 43% reported disruptions in antenatal care, and 32% reported disruptions in postnatal care (*20*). In AFR countries that provide HepB-BD, vaccination services should be maintained and disruptions in reproductive and maternal care should be addressed. In addition, more AFR countries need to consider introducing HepB-BD in routine newborn immunizations to minimize the number of new chronic HBV infections and related deaths (*18*).

When routine immunization services are adversely affected and doses are missed, a catch-up vaccination strategy is essential to complete at least 3 doses of the HBV vaccine. If HepB-BD is missed within the first 24 hours after birth, infants should be given the first dose of HBV vaccine promptly upon first contact with the health system, although effectiveness in preventing mother-to-child transmission might be reduced (*24*). WHO has developed guidance for national immunization programs to assist in establishing or refining catch-up vaccination strategies and designing catch-up vaccination schedules (*25*). HBV catch-up vaccinations can be provided through fixed, outreach, mobile, or routine school-based immunization services (*25*). Periodic campaign-style intensification of routine immunization should be considered for catchup vaccinations or sustainment of routine immunization (*21*,*26*). Communication and community engagement strategies that regularly educate communities on the availability of immunization services, need to vaccinate even if late, and COVID-19 safety measures at vaccination sites are critical for reestablishing vaccine demand and uptake (*21*,*25*). Where immunization services have been restored, countries will need to plan for potential future COVID-19 spikes and recurrent disruptions of timely vaccination schedules (*21*).

The first limitation of our study is that estimates of decreased vaccination coverage in each region were based on preliminary data at an early stage of the COVID-19 pandemic. As countries adapt immunization practices and complete data become available, our estimations may not reflect actual effects of COVID-19 on HBV vaccination coverage. However, our findings show that immunization is critical for continued progress toward the elimination of HBV. Second, coverage data from 2019 was used to estimate the excess death and chronic HBV infection in 2020. In some countries, coverage might have fluctuated before 2019, and an average coverage for the past several years could be used as an alternative baseline estimate. Third, we considered the decrease in birth dose and third dose coverage to be independent. In some countries, particularly in the WPR, HepB-BD was considered the first dose of the HBV vaccine series, and a decrease in HepB-BD would also affect third dose coverage. In other countries, particularly in the AFR, a pentavalent vaccine for the 3 additional doses was available and HepB-BD was not counted in the coverage of the third dose. Therefore, an analysis of different vaccine combinations may yield different results. Last, HBV seroprevalence data were not available from all countries, and we used data from countries with similar epidemiology when available.

The predicted consequences of COVID-19–related reduction in HBV vaccination indicate an urgent need to maintain immunization services, implement catch-up vaccinations, and mitigate disruptions in vaccination services for future births, especially in countries with a high prevalence of hepatitis B. The effects of COVID-19-related disruptions to immunization services are likely not limited to children born during 2020. It will be crucial for hospitals to include HepB-BD in their prevention and control protocols during the COVID-19 pandemic to ensure infants receive HepB-BD within 24 hours of birth. A catch-up vaccination strategy for completion of at least 3 HBV vaccine doses will be essential for children who missed their vaccinations. Countries must reduce the continued strain of COVID-19 on routine immunization services and effects on coverage that might threaten progress toward achieving hepatitis B elimination by 2030.

Appendix Additional information for effects of decreased immunization coverage for hepatitis B virus caused by COVID-19 in World Health Organization Western Pacific and African Regions, 2020.
