# Supplementary material for: Effects of Decreased Immunization Coverage for Hepatitis B Virus Caused by COVID-19 in World Health Organization Western Pacific and African Regions, 2020
Source: Emerg Infect Dis. 2022 Dec;28(Suppl 1):S217–24. doi: 10.3201/eid2813.212300 (PMC9745226; doi:10.3201/eid2813.212300)
Supplement: Appendix — Additional information for effects of decreased immunization coverage for hepatitis B virus caused by COVID-19 in World Health Organization Western Pacific and African Regions, 2020. [file 21-2300-Techapp-s1.pdf]

Article DOI: https://doi.org/10.3201/eid2813.212300

Effects of Decreased Immunization Coverage for Hepatitis B Virus Caused by COVID-19 in World Health Organization Western Pacific and African Regions, 2020

Appendix

Appendix Table 1. Prevalence of hepatitis B virus seromarkers used in the mathematical model to determine effects of decreased immunization coverage for hepatitis B virus caused by COVID-19 in World Health Organization Western Pacific and African Regions, 2020*

Country name	HBsAg prevalence among women of childbearing age (%)	HBeAg prevalence among women of childbearing age (%)	Anti-HBcAg prevalence at 5 y of age (%)	Anti-HBcAg prevalence at >30 y of age (%)	References
Africa Region					
Algeria	1.6	15	25	77.5	(*1*,*2*)
Angola	11.5	15	25	77.5	(*2*)
Benin	15.5	11.4	25	77.5	(*2*,*3*)
Botswana	11.84	15	25	77.5	(*2*)
Burkina Faso	8.1	21.2	25	77.5	(*2*,*3*)
Burundi	11.84	15	25	77.5	(*2*)
Cabo Verde	11.5	15	25	77.5	(*2*)
Cameroon	6	12.1	25	77.5	(*2*–*4*)
Central African Republic	11.84	15	25	77.5	(*2*)
Chad	11.5	15	25	77.5	(*2*)
Comoros	11.5	15	25	77.5	(*2*)
Congo	11.84	15	25	77.5	(*2*)
Côte d’Ivoire	8	14.5	25	77.5	(*2*,*3*)
The Democratic Republic of the Congo	11.84	15	25	77.5	(*2*)
Equatorial Guinea	11.5	15	25	77.5	(*2*)
Eritrea	11.84	15	25	77.5	(*2*)
Eswatini	11.84	15	25	77.5	(*2*)
Ethiopia	3.6	12.5	25	77.5	(*2*,*3*,*5*)
Gabon	9.2	10.1	25	77.5	(*2*,*3*)
Gambia	11.5	15	25	77.5	(*2*)
Ghana	12	15	25	77.5	(*2*,*3*)
Guinea	11.5	15	25	77.5	(*2*)
Guinea-Bissau	11.5	15	25	77.5	(*2*)
Kenya	9.3	8.8	25	77.5	(*2*,*3*)
Lesotho	11.84	15	25	77.5	(*2*)
Liberia	11.5	15	25	77.5	(*2*)
Madagascar	1.9	5	25	77.5	(*2*,*3*)
Malawi	11.84	15	25	77.5	(*2*)
Mali	11.8	15	25	77.5	(*2*,*3*)
Mauritania	10.7	15	25	77.5	(*2*,*3*)
Mauritius	11.5	15	25	77.5	(*2*)
Mozambique	11.84	15	25	77.5	(*2*)
Namibia	11.84	15	25	77.5	(*2*)
Niger	16.2	15	25	77.5	(*2*,*3*)
Nigeria	6.1	28.5	25	77.5	(*2*,*3*,*6*)
Rwanda	1.3	15	25	77.5	(*2*,*7*)
Sao Tome and Principe	11.84	15	25	77.5	(*2*)
Senegal	11.5	15	25	77.5	(*2*)
Seychelles	11.5	15	25	77.5	(*2*)
Sierra Leone	9.8	9.3	25	77.5	(*2*,*8*)
South Africa	3.9	17.1	25	77.5	(*2*,*3*)

Country name	HBsAg prevalence among women of childbearing age (%)	HBeAg prevalence among women of childbearing age (%)	Anti-HBcAg prevalence at 5 y of age (%)	Anti-HBcAg prevalence at >30 y of age (%)	References
South Sudan	11.5	15	25	77.5	(*2*)
Togo	11.5	15	25	77.5	(*2*)
Uganda	3	14.9	25	77.5	(*2*,*3*,*9*)
United Republic of Tanzania	3.7)	12	25	77.5	(*2*,*3*,*10*)
Zambia	4.1	15	25	77.5	(*2*,*11*)
Zimbabwe	14.2	3.3	25	77.5	(*2*,*3*)
Western Pacific Region					
American Samoa	2.55	15	26	49	(*2*,*12*,*13*)
Australia	1.28	29	0.6	11.9	(*14*,*15*,*16*)
Palau	13	40	48	58	(*17*)
Brunei Darussalam	1.1	20	25	77.5	(*2*,*18*)
Cambodia	4.39	30	25	77.5	(*2*,*19*)
China	5.44	30	32	55	(*20*,*21*)
Cook Islands	1.1	15	0.64	5.5	(*2*)
Fiji	6.6	30	25	77.5	(*2*,*22*)
French Polynesia	1.1	15	0.64	5.5	(*2*)
Guam	1.1	15	0.64	5.5	(*2*)
Hong Kong SAR, China	3.4	30	25	77.5	(*2*,*23*)
Japan	0.23	15	0.64	5.5	(*2*,*24*)
Kiribati	9.2	30	25	77.5	(*2*,*25*)
Republic of Korea	2.34	30	25	77.5	(*2*,*26*)
Lao People’s Democratic Republic	3.53	30	25	77.5	(*2*,*27*)
Malaysia	4	20	25	77.5	(*2*,*28*)
Macao SAR, China	3.4	30	25	77.5	(*2*,*23*)
Federated States of Micronesia	6.8	15	10	56.8	(*2*,*29*)
Mongolia	9.7	30	25	77.5	(*2*,*30*)
Marshall Islands	9.5	30	25	77.5	(*2*,*29*)
New Caledonia	3.3	30	25	77.5	(*2*,*31*)
New Zealand	1.1	15	0.64	5.5	(*2*)
Niue	1.1	15	0.64	5.5	(*2*)
Mariana Islands	1.1	15	0.64	5.5	(*2*)
Nauru	1.1	15	0.64	5.5	(*2*)
Philippines	18.1	20	25	77.5	(*2*,*32*)
Papua New Guinea	12.81	30	25	77.5	(*2*,*33*)
Singapore	3.7	20	25	77.5	(*2*,*34*)
Samoa	11.83	30	25	77.5	(*2*)
Solomon Islands	13.8	36.7	25	77.5	(*2*,*35*)
Tokelau	1.1	15	0.64	5.5	(*2*)
Tonga	18.6	47.5	25	77.5	(*2*,*22*)
Tuvalu	11.83	30	25	77.5	(*2*)
Vanuatu	7	30	25	77.5	(*2*,*36*)
Viet Nam	12.6	42.1	25	77.5	(*2*,*37*)
Wallis and Futuna	11.83	30	25	77.5	(*2*)

*HBcAg, hepatitis B c antigen; HBeAg, hepatitis B e antigen; HBsAg, hepatitis B surface antigen

Appendix Table 2. Number of chronic hepatitis B infections and-related deaths by country in 2019 compared with model estimates for children born in 2020 after decreased HBV immunization coverage caused by COVID-19 in the World Health Organization Western Pacific Region*

	Baseline		10% decrease in HepB- BD		20% decrease in HepB-BD		10% decrease in HepB3		20% decrease in HepB3	
Countries and areas	Infections	HBV-related deaths	Excess infections	Excess deaths	Excess infections	Excess deaths	Excess infections	Excess deaths	Excess Infections	Excess deaths
American Samoa	34	5	1 (2%)	0 (0%)	1 (4%)	0 (0%)	9 (27%)	1 (27%)	18 (54%)	3 (54%)
Australia†	1,440	344	–	–	–	–	209 (14%)	54 (16%)	417 (29%)	107 (31%)
Brunei Darussalam	39	7	1 (4%)	0 (0%)	3 (8%)	1 (7%)	61 (155%)	11 (155%)	121 (310%)	22 (310%)
Cambodia	5,277	924	396 (8%)	66 (7%)	792 (15%)	133 (14%)	3,122 (59%)	551 (60%)	6,244 (118%)	1,101 (119%)
China	123,186	25,077	24,422 (20%)	4,887 (19%)	48,844 (40%)	9,773 (39%)	157,468 (128%)	32,185 (128%)	314,935 (256%)	64,371 (257%)
Cook Islands	0	0	0 (0%)	0 (0%)	0 (0%)	0 (0%)	0 (0%)	0 (0%)	0 (0%)	0 (0%)
Fiji	129	20	34 (27%)	5 (26%)	69 (54%)	10 (51%)	169 (131%)	27 (133%)	338 (263%)	54 (265%)
French Polynesia	2	0	1 (41%)	0 (0%)	2 (81%)	0 (0%)	2 (77%)	0 (0%)	3 (153%)	1 (158%)
Guam	2	0	1 (23%)	0 (0%)	1 (46%)	0 (0%)	1 (37%)	0 (0%)	2 (74%)	0 (0%)
Hong Kong, China	897	224	76 (9%)	18 (8%)	152 (17%)	37 (17%)	754 (84%)	189 (84%)	1,508 (168%)	378 (169%)
Japan‡	666	144	–	–	–	–	399 (60%)	90 (62%)	798 (120%)	180 (125%)
Kiribati	37	7	8 (22%)	1 (21%)	16 (44%)	3 (42%)	27 (72%)	5 (72%)	53 (144%)	10 (145%)
Lao People's Democratic Republic	6,560	1,122	90 (1%)	15 (1%)	179 (3%)	29 (3%)	1,048 (16%)	180 (16%)	2,095 (32%)	361 (32%)
Macao SAR, China	52	13	7 (13%)	2 (12%)	13 (25%)	3 (25%)	65 (124%)	16 (124%)	130 (248%)	32 (249%)
Malaysia	4,576	846	466 (10%)	83 (10%)	931 (20%)	165 (20%)	5,026 (110%)	932 (110%)	10,053 (220%)	1,864 (220%)
Mariana Islands	1	0	0 (0%)	0 (0%)	0 (0%)	0 (0%)	0 (0%)	0 (0%)	1 (51%)	0 (0%)
Marshall Islands	31	5	4 (12%)	1 (11%)	7 (23%)	1 (22%)	10 (32%)	2 (32%)	20 (64%)	3 (65%)
Micronesia (Federated States of)	39	5	2 (6%)	0 (0%)	5 (12%)	1 (11%)	11 (28%)	2 (29%)	22 (56%)	3 (57%)
Mongolia	630	107	200 (32%)	33 (31%)	401 (64%)	65 (61%)	650 (103%)	112 (104%)	1,299 (206%)	223 (209%)
Nauru	0	0	0 (0%)	0 (0%)	0 (0%)	0 (0%)	0 (0%)	0 (0%)	0 (0%)	0 (0%)
New Caledonia	44	9	4 (8%)	1 (8%)	7 (16%)	1 (15%)	34 (78%)	7 (79%)	69 (157%)	15 (157%)
New Zealand‡	109	22	–	–	–	–	21 (20%)	5 (21%)	43 (39%)	9 (41%)
Niue	0	0	0 (0%)	0 (0%)	0 (0%)	0 (0%)	0 (0%)	0 (0%)	0 (0%)	0 (0%)
Palau	3	0	1 (38%)	0 (0%)	2 (76%)	0 (0%)	3 (102%)	0 (0%)	5 (205%)	1 (205%)
Papua New Guinea	20,888	3,147	212 (1%)	31 (1%)	425 (2%)	61 (2%)	701 (3%)	108 (3%)	1,402 (7%)	216 (7%)
Philippines	122,717	20,260	4,310 (4%)	687 (3%)	8,620 (7%)	1,374 (7%)	12,121 (10%)	2,045 (10%)	24,241 (20%)	4,091 (20%)
Republic of Korea	2,892	696	224 (8%)	52 (8%)	448 (15%)	105 (15%)	3,466 (120%)	837 (120%)	6,933 (240%)	1,675 (241%)
Samoa	264	49	11 (4%)	2 (4%)	21 (8%)	4 (8%)	24 (9%)	5 (9%)	49 (18%)	9 (19%)
Singapore	513	114	37 (7%)	8 (7%)	75 (15%)	16 (14%)	471 (92%)	105 (92%)	942 (184%)	210 (184%)
Solomon Islands	592	115	65 (11%)	12 (11%)	130 (22%)	25 (22%)	170 (29%)	34 (30%)	341 (58%)	68 (59%)
Tokelau	0	0	0 (0%)	0 (0%)	0 (0%)	0 (0%)	0 (0%)	0 (0%)	0 (0%)	0 (0%)
Tonga	23	5	19 (80%)	4 (78%)	37 (159%)	7 (155%)	18 (79%)	4 (81%)	37 (157%)	7 (161%)
Tuvalu	4	1	1 (24%)	0 (0%)	2 (47%)	0 (0%)	2 (57%)	0 (0%)	5 (115%)	1 (116%)
Vanuatu	165	28	14 (9%)	2 (8%)	29 (17%)	5 (17%)	73 (44%)	13 (45%)	146 (89%)	25 (90%)
Viet Nam	40,359	8,921	5,730 (14%)	1,238 (14%)	11,460 (28%)	2,476 (28%)	11,662 (29%)	2,630 (29%)	23,323 (58%)	5,260 (59%)
Wallis and Futuna	9	2	0 (0%)	0 (0%)	0 (0%)	0 (0%)	2 (22%)	0 (0%)	4 (44%)	1 (45%)
Grand total, WPR	332,179	62,222	36,342 (11%)	7,149 (11%)	72,684 (22%)	14,299 (23%)	197,797 (60%)	40,151 (65%)	395,595 (119%)	80,301 (129%)

*Values are numbers for 2019 baseline data or number (%) of infections or deaths for children born in 2020 that were in excess of 2019 values. HBV, hepatitis B virus; HepB-BD, hepatitis B birth dose vaccination; HepB3, third-dose hepatitis B vaccination.
†Australia provides HepB-BD to all newborn infants but did not report HepB-BD coverage for 2019 to the World Health Organization.
‡Japan and New Zealand provide HepB-BD only to infants born to mothers who are positive for hepatitis B surface antigen; therefore, HepB-BD coverage in 2020 was not calculated for these countries.

Appendix Table 3. Number of chronic hepatitis B infections and-related deaths by country in 2019 compared with model estimates for children born in 2020 after decreased HBV immunization coverage caused by COVID-19 in the World Health Organization African Region*

	Baseline		10% decrease in HepB-BD		20% decrease in HepB-BD		10% decrease in HepB3		20% decrease in HepB3	
Countries and areas	Infections	HBV-related deaths	Excess infections	Excess deaths	Excess infections	Excess deaths	Excess infections	Excess deaths	Excess infections	Excess deaths
Central Africa										
Angola	72,045	9,228	1,579 (2%)	190 (2%)	3,157 (4%)	379 (4%)	6,051 (8%)	785 (9%)	12,102 (17%)	1,570 (17%)
Burundi†	14,985	1,873	–	–	–	–	3,684 (25%)	486 (26%)	7,368 (49%)	972 (52%)
Cameroon†	40,685	4,812	–	–	–	–	5,519 (14%)	665 (14%)	11,039 (27%)	1,330 (28%)
Chad†	45,628	5,067	–	–	–	–	2,831 (6%)	325 (6%)	5,662 (12%)	650 (13%)
Equatorial Guinea	2,617	316	40 (2%)	4 (1%)	79 (3%)	9 (3%)	207 (8%)	25 (8%)	414 (16%)	51 (16%)
Gabon†	3,159	424	-	-	-	-	434 (14%)	59 (14%)	867 (27%)	119 (28%)
Sao Tome and Principe	78	12	14 (18%)	2 (17%)	28 (36%)	4 (34%)	58 (75%)	9 (76%)	117 (149%)	18 (151%)
Central African Republic†	11,907	1,222	–	–	–	–	665 (6%)	70 (6%)	1,330 (11%)	141 (12%)
Democratic Republic of the Congo†	227,219	30,169	–	–	–	–	17,493 (8%)	2,391 (8%)	34,986 (15%)	4,783 (16%)
Republic of the Congo†	8,163	1,067	–	–	–	–	1,250 (15%)	169 (16%)	2,501 (31%)	338 (32%)
Total	426,486	54,189	1,632 (0%)	196 (0%)	3,264 (1%)	392 (1%)	38,193 (9%)	4,986 (9%)	76,386 (18%)	9,971 (18%)
East and southern Africa										
Botswana	890	135	89 (10%)	13 (10%)	178 (20%)	26 (19%)	477 (54%)	74 (55%)	953 (107%)	148 (110%)
Eritrea†	3,330	451	–	–	–	–	892 (27%)	126 (28%)	1,783 (54%)	252 (56%)
Eswatini†	1,066	119	–	–	–	–	236 (22%)	28 (23%)	473 (44%)	55 (47%)
Ethiopia†	150,025	21,683	–	–	–	–	23,240 (15%)	3,391 (16%)	46,479 (31%)	6,783 (31%)
Kenya†	39,233	5,151	–	–	–	–	12,816 (33%)	1,742 (34%)	25,632 (65%)	3,483 (68%)
Lesotho†	2,112	203	–	–	–	–	420 (20%)	43 (21%)	839 (40%)	85 (42%)
Madagascar†	24,719	3,240	–	–	–	–	6,810 (28%)	898 (28%)	13,620 (55%)	1,795 (55%)
Malawi†	20,369	2,664	–	–	–	–	5,441 (27%)	747 (28%)	10,882 (53%)	1,494 (56%)
Mauritius‡	384	64	–	–	–	–	114 (30%)	20 (31%)	229 (60%)	40 (62%)
Mozambique†	43,150	5,223	–	–	–	–	8,900 (21%)	1,127 (22%)	17,800 (41%)	2,254 (43%)
Namibia	1,523	197	123 (8%)	15 (8%)	246 (16%)	30 (15%)	550 (36%)	72 (37%)	1,100 (72%)	145 (73%)
Rwanda†	3,799	575	–	–	–	–	3,781 (100%)	580 (101%)	7,561 (199%)	1,161 (202%)
Seychelles†	44	7	–	–	–	–	14 (32%)	2 (34%)	29 (65%)	5 (67%)
South Africa†	41,050	5,357	–	–	–	–	8,596 (21%)	1,138 (21%)	17,193 (42%)	2,277 (43%)
South Sudan†	27,061	3,252	–	–	–	–	1,632 (6%)	202 (6%)	3,263 (12%)	403 (12%)
Uganda†	28,670	3,766	–	–	–	–	14,564 (51%)	1,956 (52%)	29,127 (102%)	3,913 (104%)
United Republic of Tanzania†	46,481	6,283	–	–	–	–	18,036 (39%)	2,485 (40%)	36,073 (78%)	4,970 (79%)
Zambia†	15,601	2,071	–	–	–	–	5,363 (34%)	727 (35%)	10,725 (69%)	1,455 (70%)
Zimbabwe†	12,758	1,319	–	–	–	–	3,557 (28%)	384 (29%)	7,114 (56%)	768 (58%)
Comoros†	920	124	–	–	–	–	215 (23%)	30 (24%)	431 (47%)	60 (49%)
Total	463,185	61,884	212 (0%)	28 (0%)	424 (0%)	56 (0%)	115,654 (25%)	15,772 (25%)	231,307 (50%)	31,545 (51%)
West Africa										
Algeria	14,083	2,652	391 (3%)	70 (3%)	781 (6%)	141 (5%)	8,827 (63%)	1,664 (63%)	17,655 (125%)	3,327 (125%)
Benin	21,516	2,782	0 (0%)	0 (0%)	0 (0%)	0 (0%)	2,778 (13%)	375 (13%)	5,556 (26%)	750 (27%)
Burkina Faso†	24,212	3,273	–	–	–	–	6,215 (26%)	879 (27%)	12,430 (51%)	1,758 (54%)

	Baseline		10% decrease in HepB-BD		20% decrease in HepB-BD		10% decrease in HepB3		20% decrease in HepB3	
Countries and areas	Infections	HBV-related deaths	Excess infections	Excess deaths	Excess infections	Excess deaths	Excess infections	Excess deaths	Excess infections	Excess deaths
Cabo Verde	99	16	21 (21%)	3 (20%)	42 (43%)	7 (41%)	93 (93%)	15 (94%)	185 (186%)	31 (188%)
Côte d'Ivoire	29,963	3,353	116 (0%)	12 (0%)	233 (1%)	25 (1%)	6,915 (23%)	802 (24%)	13,830 (46%)	1,604 (48%)
Gambia	2,711	344	66 (2%)	8 (2%)	132 (5%)	16 (5%)	706 (26%)	93 (27%)	1,412 (52%)	186 (54%)
Ghana†	26,983	3,458	–	–	–	–	7,686 (28%)	1,041 (30%)	15,372 (57%)	2,082 (60%)
Guinea†	33,928	4,380	–	–	–	–	1,919 (6%)	254 (6%)	3,838 (11%)	507 (12%)
Guinea-Bissau†	2,681	303	–	–	–	–	489 (18%)	58 (19%)	978 (36%)	116 (38%)
Liberia†	8,044	1,102	–	–	–	–	1,065 (13%)	151 (14%)	2,130 (26%)	301 (27%)
Mali†	37,884	4,715	–	–	–	–	5,403 (14%)	702 (15%)	10,806 (29%)	1,403 (30%)
Mauritania§	6,338	884	0 (0%)	0 (0%)	0 (0%)	0 (0%)	1,090 (17%)	157 (18%)	2,179 (34%)	315 (36%)
Nigeria	384,442	47,258	6,402 (2%)	725 (2%)	12,804 (3%)	1,450 (3%)	37,659 (10%)	4,704 (10%)	75,318 (20%)	9,408 (20%)
Senegal	9,051	1,328	952 (11%)	134 (10%)	1,905 (21%)	268 (20%)	4,700 (52%)	703 (53%)	9,400 (104%)	1,406 (106%)
Sierra Leone†	5,968	591	–	–	–	–	2,123 (36%)	223 (38%)	4,245 (71%)	446 (76%)
Togo†	10,810	1,336	–	–	–	–	1,971 (18%)	255 (19%)	3,942 (36%)	509 (38%)
Niger†	56,304	7,321	–	–	–	–	7,539 (13%)	1,031 (14%)	15,078 (27%)	2,062 (28%)
Total	675,017	85,096	7,949 (1%)	953 (1%)	15,897 (2%)	1,906 (2%)	97,177 (14%)	13,106 (15%)	194,354 (29%)	26,211 (31%)
Grand Total, African Region	1,564,688	201,170	9,792 (1%)	1,177 (1%)	19,585 (1%)	2,355 (1%)	251,023 (16%)	33,864 (17%)	502,047 (32%)	67,727 (34%)

*Values are numbers for 2019 baseline data or number (%) of infections or deaths for children born in 2020 that were in excess of 2019 values. HBV, hepatitis B virus; HepB-BD, hepatitis B birth dose vaccination; HepB3, third-dose hepatitis B vaccination.
†These countries do not provide HepB-BD; therefore, coverage was not applicable.
‡Mauritius provides HepB-BD only to infants born to mothers who are positive for hepatitis B surface antigen; therefore, HepB-BD coverage in 2020 was not calculated for these countries.
§Mauritania provides HepB-BD to all newborn infants; however, no coverage was reported for HepB-BD vaccinations within the first 24 h after birth in 2019.

References

1. Madihi S, Syed H, Lazar F, Zyad A, Benani A. A systematic review of the current hepatitis B viral infection and hepatocellular carcinoma situation in Mediterranean countries. BioMed Res Int. 2020;2020:7027169. PubMed https://doi.org/10.1155/2020/7027169

2. Goldstein ST, Zhou F, Hadler SC, Bell BP, Mast EE, Margolis HS. A mathematical model to estimate global hepatitis B disease burden and vaccination impact. Int J Epidemiol. 2005;34:1329–39. PubMed https://doi.org/10.1093/ije/dyi206

3. Breakwell L, Tevi-Benissan C, Childs L, Mihigo R, Tohme R. The status of hepatitis B control in the African region. Pan Afr Med J. 2017;27:17. PubMed https://doi.org/10.11604/pamj.supp.2017.27.3.11981

4. Republic of Cameroon Ministry of Health, Division of Health Operations Research. Cameroon population-based HIV impact assessment (CAMPHIA) 2017–2018: final report, Yaounde, Republic of Cameroon. December 2020 [cited 2022 Mar 25]. https://phia.icap.columbia.edu/wp-content/uploads/2021/09/53059-CAMPHIA-Report_EN_WEB_August1.pdf

5. Ethiopian Public Health Institute. Ethiopia population-based HIV impact assessment (EPHIA) 2017–2018: final report. August 2020 [cited 2022 Mar 25]. https://phia.icap.columbia.edu/wp-content/uploads/2020/11/EPHIA_Report_280820_Web.pdf

6. Federal Ministry of Health, Nigeria. Nigeria HIV/AIDS indicator and impact survey (NAIIS) 2018: technical report. October 2019 [cited 2022 Mar 25]. http://ciheb.org/media/SOM/Microsites/CIHEB/documents/NAIIS-Report-2018.pdf

7. Rwanda Biomedical Center. Rwanda population-based HIV impact assessment (RPHIA) 2018–2019: final report. September 2020 [cited 2022 Mar 25]. https://www.rbc.gov.rw/fileadmin/user_upload/report2019/53059%20RPHIA_Report_V9_Web.pdf

8. Breakwell L, Marke D, Kaiser R, Tejada-Strop A, Pauly MD, Jabbi S, et al. Assessing the impact of the routine childhood hepatitis B immunization program and the need for hepatitis B vaccine birth dose in Sierra Leone, 2018. Vaccine. 2022;40:2741–8. PubMed https://doi.org/10.1016/j.vaccine.2022.03.049

9. Ministry of Health, Uganda. Uganda population-based HIV impact assessment (UPHIA) 2016–2017: final report. July 2019 [cited 2022 Mar 25]. https://phia.icap.columbia.edu/wp-content/uploads/2020/02/UPHIA_Final_Report_Revise_07.11.2019_Final_for-web.pdf

10. Tanzania Commission for AIDS, Zanzibar AIDS Commission. Tanzania HIV impact survey (THIS) 2016–2017: final report. December 2018 [cited 2022 Mar 25]. https://phia.icap.columbia.edu/wp-content/uploads/2019/06/FINAL_THIS-2016-2017_Final-Report__06.21.19_for-web_TS.pdf

11. Ministry of Health, Zambia. Zambia population-based HIV impact assessment (ZAMPHIA) 2016: final report. February 2019 [cited 2022 Mar 25] https://phia.icap.columbia.edu/wp-content/uploads/2019/03/ZAMPHIA-Final-Report__2.26.19.pdf

12. Koneru A, Schillie S, Roberts H, Sirotkin B, Fenlon N, Murphy TV, et al. Estimating annual births to hepatitis B surface antigen-positive women in the United States by using data on maternal country of birth. Public Health Rep. 2019;134:255–63. PubMed https://doi.org/10.1177/0033354919836958

13. Mahoney FJ, Woodruff BA, Erben JJ, Coleman PJ, Reid EC, Schatz GC, et al. Effect of a hepatitis B vaccination program on the prevalence of hepatitis B virus infection. J Infect Dis. 1993;167:203–7. PubMed https://doi.org/10.1093/infdis/167.1.203

14. Cheng EH, Witharana S, Haque M. The impact of maternal chronic hepatitis B infection in obstetric outcomes [abstract]. In: 23rd Annual Conference of APASL March 12–15, 2014, Brisbane, Queensland, Australia. Hepatol Int. 2014;8:S147–8. https://doi.org.10.1007/s12072-014-9519-7

15. Wiseman E, Fraser MA, Holden S, Glass A, Kidson BL, Heron LG, et al. Perinatal transmission of hepatitis B virus: an Australian experience. Med J Aust. 2009;190:489–92. PubMed https://doi.org/10.5694/j.1326-5377.2009.tb02524.x

16. O'Sullivan BG, Gidding HF, Law M, Kaldor JM, Gilbert GL, Dore GJ. Estimates of chronic hepatitis B virus infection in Australia, 2000. Aust N Z J Public Health. 2004;28:212–6. PubMed https://doi.org/10.1111/j.1467-842X.2004.tb00697.x

17. Mahoney FJ, Woodruff B, Auerbach S, Polloi A, McCready J, Durand M, et al. Progress on the elimination of hepatitis B virus transmission in Micronesia and American Samoa. Pac Health Dialog. 1996;3:140–6. https://www.pacifichealthdialog.org.fj/Volume203/No220Communicable20diseases20and20Environmental20Health20in20the20Pacific/Original20Papers/PROGR201C.PDF

18. Htwe O, Coates PD, Krasu M, Tju H, Soe NN, Tan C, et al. Prevalence of hepatitis B and other infections among pregnant women seen in a referral centre in Brunei Darussalam. Brunei Int Med J. 2013;9:220–26. https://eprints.usq.edu.au/24245/1/Htwe_etal_BIMJ_v9n4_PV.pdf

19. Ork V, Woodring J, Shafiqul Hossain M, Wasley A, Nagashima S, Yamamoto C, et al. Hepatitis B surface antigen seroprevalence among pre- and post-vaccine cohorts in Cambodia, 2017. Vaccine. 2019;37:5059–66. PubMed https://doi.org/10.1016/j.vaccine.2019.06.073

20. Liu J, Wang X, Wang Q, Qiao Y, Jin X, Li Z, et al. Hepatitis B virus infection among 90 million pregnant women in 2853 Chinese counties, 2015–2020: a national observational study. Lancet Reg Health West Pac. 2021;16:100267. PubMed https://doi.org/10.1016/j.lanwpc.2021.100267

21. Cui F, Shen L, Li L, Wang H, Wang F, Bi S, et al. Prevention of chronic hepatitis B after 3 decades of escalating vaccination policy, China. Emerg Infect Dis. 2017;23:765–72. PubMed https://doi.org/10.3201/eid2305.161477

22. Wilson N, Ruff TA, Rana BJ, Leydon J, Locarnini S. The effectiveness of the infant hepatitis B immunisation program in Fiji, Kiribati, Tonga and Vanuatu. Vaccine. 2000;18:3059–66. PubMed https://doi.org/10.1016/s0264-410x(00)00080-3

23. Hong Kong Department of Health, Viral Hepatitis Control Office. Mother-to-child transmission of hepatitis B [cited 2022 Mar 25]. https://www.hepatitis.gov.hk/english/mtct/maternal_transmission_of_hepatitis_b.html

24. Sugiyama A, Yamashita M, Ko K, Ohisa M, Akita T, Wakita T, et al. Epidemiological assessment of interventions to eliminate mother-to-child transmission of hepatitis B virus in Japan. GastroHep. 2021;3:72–9. https://doi.org/10.1002/ygh2.441

25. World Health Organization. Prevalence surveys of sexually transmitted infections among seafarers and women attending antenatal clinics in Kiribati: 2002–2003. 2004. https://apps.who.int/iris/handle/10665/206930

26. Kim H, Shin AR, Chung HH, Kim MK, Lee JS, Shim JJ, et al. Recent trends in hepatitis B virus infection in the general Korean population. Korean J Intern Med. 2013;28:413–9. PubMed https://doi.org/10.3904/kjim.2013.28.4.413

27. Xeuatvongsa A, Komada K, Kitamura T, Vongphrachanh P, Pathammavong C, Phounphenghak K, et al. Chronic hepatitis B prevalence among children and mothers: results from a nationwide, population-based survey in Lao People's Democratic Republic. PLoS One. 2014;9:e88829. PubMed https://doi.org/10.1371/journal.pone.0088829

28. Muhamad NA, Ab Ghani RM, Abdul Mutalip MH, Muhammad EN, Mohamad Haris H, Mohd Zain R, et al. Seroprevalence of hepatitis B virus and hepatitis C virus infection among Malaysian population. Sci Rep. 2020;10:21009. PubMed https://doi.org/10.1038/s41598-020-77813-5

29. Bialek SR, Helgenberger L, Fischer GE, Bower WA, Konelios M, Chaine JP, et al. Impact of routine hepatitis B immunization on the prevalence of chronic hepatitis B virus infection in the marshall islands and the federated states of micronesia. Pediatr Infect Dis J. 2010;29:18–22. PubMed https://doi.org/10.1097/INF.0b013e3181b20e93

30. Dashdorj N, Dashtseren B, Bold B, Yagaanbuyant D. Epidemiological study of prevalence and risk factors for HBV among apparently healthy Mongolians [abstract]. In: Viral Hepatitis Congress 2014, 9–11 October 2014, Frankfurt, Germany. J Viral Hepat. 2014;21:23–24. https://onlinelibrary.wiley.com/doi/epdf/10.1111/jvh.12333_4

31. Berlioz-Arthaud A, Perolat P, Buisson Y. 10 year assessment of infant hepatitis B vaccination program, in the Loyalty Islands (New Caledonia). Vaccine. 2003;21:2737–42. PubMed https://doi.org/:10.1016/s0264-410x(03)00226-3

32. Wong SN, Ong JP, Labio MED, Cabahug OT, Daez MLO, Valdellon EV, et al. Hepatitis B infection among adults in the Philippines: a national seroprevalence study. World J Hepatol. 2013;5:214–9. PubMed https://doi.org/10.4254/wjh.v5.i4.214

33. Lee AU, Mair L, Kevin B, Gandi L, Tarumuri O, Lee C, et al. Prevalence of chronic hepatitis B in Oro Province, Papua New Guinea. Western Pac Surveill Response J. 2020;11:6–9. PubMed https://doi.org/10.5365/wpsar.2020.11.3.001

34. Ang LW, Cutter J, James L, Goh KT. Seroepidemiology of hepatitis B virus infection among adults in Singapore: a 12-year review. Vaccine. 2013;32:103–10. PubMed https://doi.org/10.1016/j.vaccine.2013.10.057

35. Getahun A, Baekalia M, Panda N, Lee A, Puiahi E, Khan S, et al. Seroprevalence of hepatitis B surface antigen in pregnant women attending antenatal clinic in Honiara Solomon Islands, 2015. World J Hepatol. 2016;8:1521–8. PubMed https://doi.org/10.4254/wjh.v8.i34.1521

36. Natuman SL. Hepatitis B the Vanuatu story evidence to impact [conference presentation]. In: Vanuatu 2nd Health Research Symposium, 23–24 September 2021, Port Vila, Vanuatu. [cited 2022 April 6]. https://moh.gov.vu/healthsymposium/docs/presentation/Sereana%20Natuman_2%20PowerPointToPdf.pdf

37. Miyakawa M, Yoshida LM, Nguyen HT, Takahashi K, Le TH, Yasunami M, et al. Hepatitis B virus infection among pregnant mothers and children after the introduction of the universal vaccination program in Central Vietnam. Sci Rep. 2021;11:8676. PubMed https://doi.org/10.1038/s41598-021-87860-1
